# Cancer testis antigen burden (CTAB): a novel biomarker of tumor-associated antigens in lung cancer

**DOI:** 10.1186/s12967-024-04918-0

**Published:** 2024-02-07

**Authors:** R. J. Seager, Maria-Fernanda Senosain, Erik Van Roey, Shuang Gao, Paul DePietro, Mary K. Nesline, Durga Prasad Dash, Shengle Zhang, Heidi Ko, Stephanie B. Hastings, Kyle C. Strickland, Rebecca A. Previs, Taylor J. Jensen, Marcia Eisenberg, Brian J. Caveney, Eric A. Severson, Shakti Ramkissoon, Jeffrey M. Conroy, Sarabjot Pabla

**Affiliations:** 1 OmniSeq (Labcorp Oncology), Buffalo, NY USA; 2 Labcorp Oncology, Durham, NC USA; 3 Duke University Medical Center, Duke Cancer Institute, Durham, NC USA; 4 Labcorp, Burlington, NC USA; 5 Wake Forest Comprehensive Cancer Center, Wake Forest School of Medicine, Winston-Salem, NC USA

**Keywords:** Tumor microenvironment, Inflammation, Immunotherapy, Immune checkpoint inhibitors, Gene expression profiling

## Abstract

**Background:**

Cancer-testis antigens (CTAs) are tumor antigens that are normally expressed in the testes but are aberrantly expressed in several cancers. CTA overexpression drives the metastasis and progression of lung cancer, and is associated with poor prognosis. To improve lung cancer diagnosis, prognostic prediction, and drug discovery, robust CTA identification and quantitation is needed. In this study, we examined and quantified the co-expression of CTAs in lung cancer to derive cancer testis antigen burden (CTAB), a novel biomarker of immunotherapy response.

**Methods:**

Formalin fixed paraffin embedded (FFPE) tumor samples in discovery cohort (n = 5250) and immunotherapy and combination therapy treated non-small cell lung cancer (NSCLC) retrospective (n = 250) cohorts were tested by comprehensive genomic and immune profiling (CGIP), including tumor mutational burden (TMB) and the mRNA expression of 17 CTAs. PD-L1 expression was evaluated by IHC. CTA expression was summed to derive the CTAB score. The median CTAB score for the discovery cohort of 170 was applied to the retrospective cohort as cutoff for CTAB “high” and “low”. Biomarker and gene expression correlation was measured by Spearman correlation. Kaplan–Meier survival analyses were used to detect overall survival (OS) differences, and objective response rate (ORR) based on RECIST criteria was compared using Fisher’s exact test.

**Results:**

The CTAs were highly co-expressed (p < 0.05) in the discovery cohort. There was no correlation between CTAB and PD-L1 expression (R = 0.011, p = 0.45) but some correlation with TMB (R = 0.11, p = 9.2 × 10^–14^). Kaplan–Meier survival analysis of the immunotherapy-treated NSCLC cohort revealed better OS for the pembrolizumab monotherapy treated patients with high CTAB (p = 0.027). The combination group demonstrated improved OS compared to pembrolizumab monotherapy group (p = 0.04). The pembrolizumab monotherapy patients with high CTAB had a greater ORR than the combination therapy group (p = 0.02).

**Conclusions:**

CTA co-expression can be reliably measured using CGIP in solid tumors. As a biomarker, CTAB appears to be independent from PD-L1 expression, suggesting that CTAB represents aspects of tumor immunogenicity not measured by current standard of care testing. Improved OS and ORR for high CTAB NSCLC patients treated with pembrolizumab monotherapy suggests a unique underlying aspect of immune response to these tumor antigens that needs further investigation.

**Supplementary Information:**

The online version contains supplementary material available at 10.1186/s12967-024-04918-0.

## Background

In the last two decades, immunotherapy has revolutionized cancer therapy. Immune checkpoint inhibitors (ICIs) targeting the Programmed Cell Death 1 (PD-1) receptor and its ligands have proved highly effective in numerous cancer types. However, despite the success of ICIs, not all patients will derive benefit and others will develop resistance over time [1–3]. Consequently, finding ways to increase the efficacy of these drugs and identify patients who will derive the most benefit is an important theme of ongoing research [4, 5]. One important focus area in service of both goals is the identification of new targets for these drugs and biomarkers of patients likely to respond to such treatments [2, 6–9]. Specifically for immuno-oncology, multiplex immunohistochemistry and digital special profiling of tumor biopsies have revealed complex tumor-immune interactions affecting response to checkpoint inhibitors [10]. As a result, many immune-associated proteins are being explored as potential drug targets or biomarkers of treatment response [11]. One such group of proteins are cancer testis antigens (CTAs).

CTAs are a group of proteins commonly included in the tumor-associated antigens family and consist of approximately 250 proteins and associated genes [12]. CTAs are normally expressed in the testes and placenta but can also be aberrantly expressed in various types of cancer. A considerable amount of research and treatment development has centered on exploiting this restricted expression pattern by using CTAs as targets for cancer biomarkers and immunotherapies [13]. This aberrant expression emerges as a result of DNA hypomethylation and histone modifications of the promoter regions of these genes [14]. CTAs are generally divided into two groups, CT-X antigens located on the X chromosome (52%) and non-X CTAs (48%) [12]. As of 2023, more than 200 CTAs have been identified [15].

The testes and placenta, where CTA are exclusively expressed, are considered immune privileged, meaning the introduction of antigens at these sites will not induce an inflammatory immune response [16]. This lack of contact between the immune system and CTA proteins results in a recognition of these proteins as “non-self” when expressed elsewhere in the body, resulting in the inherent immunogenicity of CTAs [14, 17]. In the context of competition between tumor development and immune surveillance, the widely observed expression of CTAs across multiple tumor types must confer significant survival benefits to tumors outweighing the potential existential risk posed by the immune response to abnormal CTA expression. Indeed, there is evolving evidence showing that CTAs participate in tumorigenesis and progression by sustaining proliferative signaling, resisting cell death, evading growth suppressors, angiogenesis induction, major histocompatibility complex downregulation (immune evasion), deregulating cellular energetics, and genome instability [13, 15], and CTAs are linked to cancer drug resistance [15]. Therefore, CTAs often represent a uniquely identifying and developmentally integral aspect of tumor cell biology.

CTA-based biomarkers take advantage of this unique position of CTAs with respect to both the other, normally functioning cells in the body and the other aberrantly expressed genes found in tumor cells, and have potential applications in the diagnosis, monitoring, and treatment of cancer [18–21]. Given the restricted expression pattern and strong antigenicity of CTAs, they can serve as potential prognostic biomarkers and targets for immunotherapeutic interventions including ICIs, cancer vaccines, cellular and antibody-based therapies [18, 22]. Individual CTAs have been previously used as biomarkers for prognosis and diagnosis in multiple tumor types [23]. In particular, CTAs such as *NY-ESO-1* [24], *MAGE-1* [25], *SSX2* [26], and *LAGE-1* [27], are currently investigated as potential targets and studied as prospective biomarkers in various cancers. Therefore, CTA-based biomarkers present an additional metric by which cancer may be diagnosed and monitored, but also a valuable tool for the selection of patients likely to respond to treatments directly or indirectly targeting CTA, particularly those leveraging the immunogenic nature of these proteins.

To identify patients who may benefit from CTA-targeted therapy, CTA expression levels in patient biopsies must be detected and analyzed. The primary technologies currently available to assess CTA expression in solid tumors include immunohistochemistry (IHC) in tissue samples, various serological assays in liquid biopsy, polymerase chain reaction (PCR) and RNA-seq. However, given the considerable number of CTA expressed across many cancer types, assessing the aggregate expression of multiple CTAs with RNA-seq allow for the succinct quantification of CTA expression and co-expression.

In this study, we employ RNA-seq to assess the distribution of CTA expression and co-expression across 22 types of solid tumors and propose a biomarker of aggregate CTA expression, termed cancer testis antigen burden (CTAB). We demonstrate that CTAB measures aspects of the tumor microenvironment not assessed by traditional biomarkers of immunotherapy response, namely Programmed Death-Ligand 1 (PD-L1) expression and tumor mutational burden (TMB). Finally, we show the utility of CTAB as a biomarker of immunotherapy response in non-small cell lung cancer (NSCLC) and highlight how the immunological aspects of CTA expression in NSCLC may have implications on the design of therapeutic strategies incorporating ICIs as mono- and combination therapies.

## Methods

### Patients and clinical data

This study involves two separate cohorts, a discovery cohort of clinically tested solid tumors used for development of the immunogenic signature and a retrospective NSCLC cohort for which response to ICI therapy and overall survival was available. Both cohorts were assembled from real-world patient samples upon which comprehensive genomic and immune profiling (CGIP) was performed during the course of routine clinical care using the OmniSeq Insight assay [28, 29]. For the discovery cohort, a total of 5624 patients were included based on the following criteria: (1) availability of high-quality gene expression data from samples clinically tested by a CLIA (Clinical Laboratory Improvement Amendments) approved targeted RNA-seq assay [28, 29]; (2) samples that pass clinically approved tissue, nucleic acid and sequencing quality control metrics; (3) samples that have less than 50% necrosis and at least 5% tumor purity; and (4) availability of PD-L1 IHC and TMB (Table 1).

**Table 1:** Discovery cohort description

Variable	Group	N	% of cohort (%)
Tumor type	Adrenal gland cancer	4	0.1
	Bladder cancer	80	1.5
	Brain and nervous system cancer	55	1.1
	Breast cancer	487	9.3
	Cervical cancer	49	0.9
	Colorectal cancer	603	11.5
	Esophageal cancer	159	3.0
	Head and neck cancer	144	2.7
	Kidney and renal pelvis cancer	42	0.8
	Liver and bile duct cancer	81	1.5
	Lung cancer	2264	43.1
	Melanoma	129	2.5
	Mesothelioma	19	0.4
	Ovarian cancer	270	5.1
	Pancreatic cancer	216	4.1
	Prostate cancer	153	2.9
	Sarcoma	192	3.7
	Stomach cancer	101	1.9
	Testicular cancer	4	0.1
	Thymic cancer	11	0.2
	Thyroid cancer	32	0.6
	Uterine cancer	155	3.0
Cancer type	Metastatic	2381	45.4
	Primary	2819	53.7
	Recurrent	45	0.9
	No data	5	0.1
All samples		5250	100.0

The retrospective cohort of 250 tumors were from patients with NSCLC (213 non-squamous, 37 squamous) treated with ICIs (Table 2), as previously described [30]. The inclusion criteria for this retrospective cohort study required patients to have received treatment with an FDA-approved ICI as of November 2017, and to have follow-up and survival data from the first ICI dose. In addition, patients had to have an evaluable response based on Response Evaluation Criteria in Solid Tumors (RECIST) v1.1 criteria. Patients who had a complete response (CR) or partial response (PR) based on RECIST v1.1 criteria were classified as responders, while those who had stable disease (SD), or progressive disease (PD) were classified as non-responders. However, the duration of response was not available for all patients and was not included in the final analysis.

**Table 2:** Retrospective cohort description

Variable	Group	N	% of cohort (%)
Age	[30, 40)	1	0.4
	[40, 50)	5	2.0
	[50, 60)	40	16.0
	[60, 70)	105	42.0
	[70, 80)	75	30.0
	[80, 90)	23	9.2
	[90, 100)	1	0.4
Sex	Female	136	54.4
	Male	114	45.6
Tumor type	NSCLC	250	100.0
Histology type	Non-squamous	213	85.2
	Squamous	37	14.8
Treatment group	Pembro. + chemo	148	59.2
	Pembrolizumab	102	40.8
All samples		250	100.0

Supporting whole transcriptome data used to duplicate the derivation of CTAB biomarker in the discovery cohort was obtained from The Cancer Genome Atlas (TCGA) [31]. Whole-transcriptome tissue type-specific CTA expression data for normal tissue was obtained from GTEx (Genotype-Tissue Expression) [32] (Additional File 6, Table S1).

### Quality assessment of clinical FFPE tissue specimens

Tissue sections from formalin fixed paraffin embedded (FFPE) blocks were cut to a thickness of 5 µm onto positively charged slides. One section from each tissue sample was stained with hematoxylin and eosin and examined by a board-certified anatomical pathologist to assess the adequacy of tumor representation, presence of necrosis or issues with fixation or handling, and quality of tissue preservation. Specimens with less than 5% tumor tissue and more than 50% necrosis were excluded from analysis. To achieve the assay requirements for RNA (10 ng) and DNA (20 ng) input, tissue from 3 to 5 unstained slide sections was required, with or without tumor macrodissection.

### Nucleic acid isolation

DNA and RNA were co-extracted from each tissue sample and processed for gene expression analysis by RNA-seq and TMB analysis by DNA-seq, as previously described [28, 29]. The extracted nucleic acids were then quantified using a Qubit fluorometer (Thermo Fisher Scientific), which uses ribogreen staining for RNA and picogreen staining for DNA. PD-L1 status of each tumor was assessed by IHC.

### Genomic and immune profiling

Gene expression were evaluated by RNA sequencing of 395 transcripts on samples that met validated quality control thresholds [28, 29]. Potential pre-analytical interference to the gene expression values was assessed during assay validation as previously described [28]. TMB was measured by DNA sequencing of the full coding region of 409 cancer related genes as non-synonymous mutations per megabase (mut/Mb) of sequenced DNA on samples with > 30% tumor nuclei and TSO500 genomic profiling [29]. To perform the RNA-seq and DNA-seq analyses, libraries of the extracted nucleic acids were prepared and sequenced to appropriate depth on the Ion Torrent S5XL sequencer (Thermo Fisher Scientific) and NovaSeq 6000 (Illumina). PD-L1 expression tumor proportion score (TPS) was assessed by IHC (22C3).

### Data analyses

The RNA-seq data was processed using the Torrent Suite plugin immuneResponseRNA (Thermo Fisher Scientific), which generated absolute reads for each transcript [28]. For each gene, the expression values were then converted to a percentile rank of 0–100 when compared to a reference population of 735 solid tumors of 35 histologies [28]. Genomic profiling was performed using Illumina TSO500 analysis pipeline [Illumina: v2.1.0.60] [29]. All subsequent data analyses were performed using R (v4.3.0). To analyze the relationship between CTA gene expression and clinical outcomes, Spearman correlation (ρ or r_s_) analysis was performed. Network graph visualization of the correlations between CTAs was performed using a Fruchterman-Reingold force-directed algorithm to arrange nodes representing each CTA connected by edges weighted according to the absolute value of the Spearman correlation observed between each pair of CTAs [33]. Continuous variables were compared between patient groups using Kruskal–Wallis or Wilcoxon Rank-Sum tests as appropriate. Kaplan–Meier (KM) analysis was used for survival analysis using two-year survival data. Treatment response was compared between patient groups using Fisher’s Exact Test without continuity correction. In addition, two previously published gene expression signatures were calculated: the cell proliferation (CP) signature [34, 35] and the tumor immunogenic signature (TIGS), which measures the “hot” or “cold” inflammation state of the tumor microenvironment [36]. P-values less than 0.05 were considered to be significant. The CTAB biomarker was calculated by summing the gene expression ranks of 17 CTAs (*BAGE*, *CTAG1B* (*NY-ESO-1*), *CTAG2* (*LAGE-1A*), *GAGE1*, *GAGE10*, *GAGE12J*, *GAGE13*, *GAGE2*, *MAGEA1*, *MAGEA10*, *MAGEA12*, *MAGEA3*, *MAGEA4*, *MAGEC2*, *MLANA*, *SSX2*, and *XAGE1B*), resulting in an integer value between 0 and 1700 for each sample with gene expression data for all 17 CTAs.

## Results

### Restricted expression of CTAs in testis tissue

Data from the tissue gene expression database GTEx was profiled in order to evaluate the expression patterns of CTAs across diverse tissue types. Among the 54 tissue types profiled, robust CTA expression was observed only in testis tissue (Additional file 1: Fig. S1), in agreement with the previously reported restricted expression of CTAs [12]. Additionally, immune-associated genes such as CD8A and PD-L1 (CD274) were expressed in most of the tissue types profiled, with the notable exception of the testes, ovaries, and CNS, in line with the immuno-privileged nature of those tissue types (Additional file 1: Fig. S1) [37].

### CTA co-expression

**Fig. 1:**
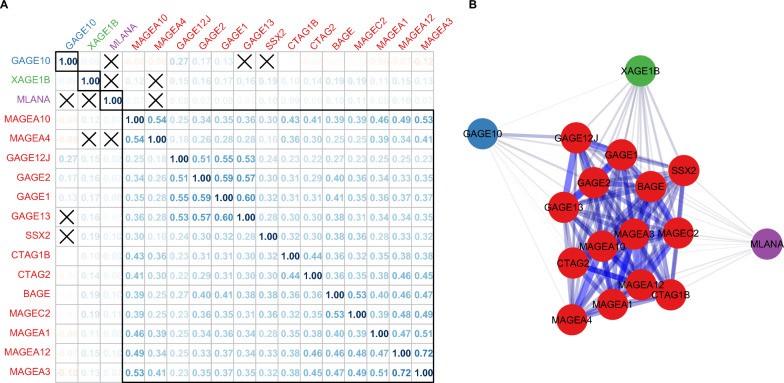
Cancer testis antigen (CTA) co-expression in the discovery cohort. **A** Correlation plot detailing the pairwise Pearson correlations of 17 CTAs. All statistically significant (p ≤ 0.05) correlations are denoted, and an “X” indicates a nonsignificant correlation. The black rectangles are visual aids indicating the four observed groups of CTA expression, and the colors of the gene labels on both axes are additional visual aids to reflect these groups. **B** Network graph of CTA co-expression in the discovery cohort where the thickness and length of each edge represent the absolute value of the correlation between two CTA (a thick, short edge denotes a strong correlation and a thin, long edge denotes a weak correlation) and the color of each edge represents the direction of each observed correlation (a red edge represents a positive correlation and a blue edge represents a negative correlation). Note that edges are only shown for significant (p ≤ 0.05) correlations. The color of each node matches the axis gene labels on the correlation plot (**a**) and correspond to the main groups of CTA expression

RNA-seq of the discovery cohort was used to determine the expression of 17 CTA genes in 5624 real-world solid tumor tissues. High levels of CTA co-expression were observed in multiple tumor types, quantified by significant positive Spearman correlation (r_s_), between the 17 CTA (Fig. 1a, b). In the discovery cohort, 14 of the 17 profiled CTA were significantly co-expressed with one another, and the remaining three were expressed largely independently of the others, resulting in four CTA expression groups: (1) *MAGEA10*, *MAGEA4*, *GAGE12J*, *GAGE2*, *GAGE1*, *GAGE13*, *SSX2*, *CTAG1B*, *CTAG2*, *BAGE*, *MAGEC2*, *MAGEA1*, *MAGEA12*, and *MAGEA3* [mean r_s,group_ = 0.37, mean r_s,all_ = 0.28, max r_s,any_ = 0.72] (2) *MLANA* [mean r_s,all_ = 0.08, max r_s,any_ = 0.10]; (3) *XAGE1B* [mean r_s,all_ = 0.14, max r_s,any_ = 0.20]; and (4) *GAGE10* [mean r_s,all_ = 0.02, max r_s,any_ = 0.27] (Fig. 1a, b). Similar co-expression was observed in the TCGA cohort (Additional file 3: Fig. S3).

### CTAB in cancer

**Fig. 2:**
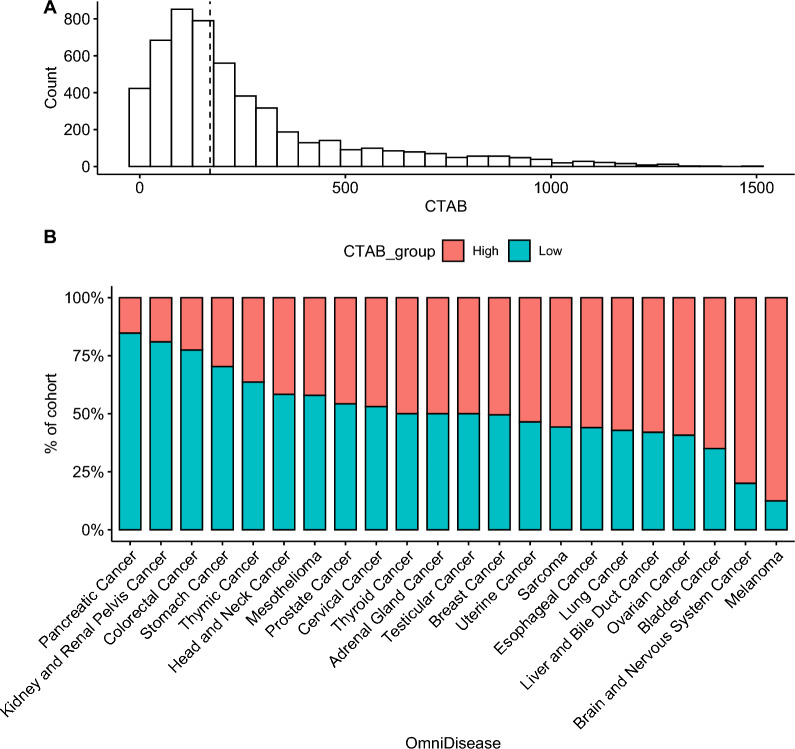
Cancer testis antigen burden (CTAB) distributions in the discovery cohort: **A** Overall CTAB distribution (median of 170 shown, **B**) distributions of CTAB for 22 tumor types in the discovery cohort

To capture this combined CTA expression, CTAB was calculated by summing the gene expression rank of all 17 CTAs in the discovery cohort, giving an integer value between 0 and 1700. The median CTAB for the discovery cohort was 170. Analysis of CTAB in 22 distinct types of tumors revealed different mean CTAB among the tumors evaluated (Fig. 2b). The highest CTAB distribution was observed in melanoma and lowest in colorectal cancer (Fig. 2b). Similar observations were made in an independently assembled TCGA cohort (Additional file 3: Fig. S3a, b).

### Biomarker correlations with CTAB

**Fig. 3:**
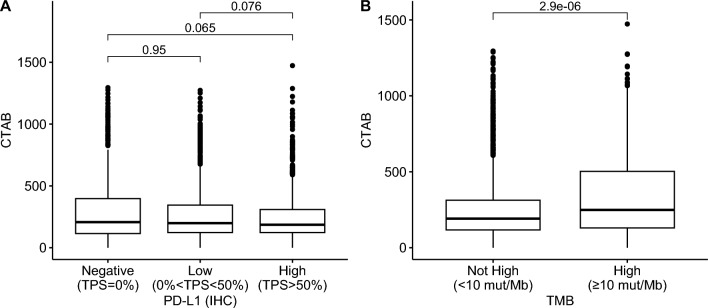
Box plots of the cancer testis antigen burden (CTAB) score: **A** across PD-L1 groups, as measured by immunohistochemistry (IHC) and (**B**) across tumor mutational burden (TMB) groups. Statistical comparisons between groups are indicated with Wilcoxon Rank-Sum p-values

We investigated the statistical relationship between CTAB and previously established biomarkers of response to checkpoint inhibition in solid tumors. When the discovery cohort was subdivided by PD-L1 classification by IHC, no significant difference in CTAB was observed between the negative (TPS = 0%), low (0% < TPS < 50%), and high (TPS ≥ 50%) subgroups (Fig. 3a). When the discovery cohort was similarly subdivided based on TMB, a significant difference was observed between TMB < 10 mut/Mb and TMB ≥ 10 mut/Mb subgroups (p = 2.9 × 10^–6^).

The correlations between CTAB and other immune biomarkers including PD-L1 assessed by RNA-seq and two additional gene expression signatures, tumor immunogenic score (TIGS) and cell proliferation (CP), were also assessed in both the discovery cohort and the TCGA cohort (Additional file 4: Fig. S4). These analyses revealed weak or nonsignificant correlations between CTAB and these other biomarkers, suggesting that CTAB measures aspects of the tumor microenvironment independent from these biomarkers.

### NSCLC survival and treatment response analyses

**Fig. 4:**
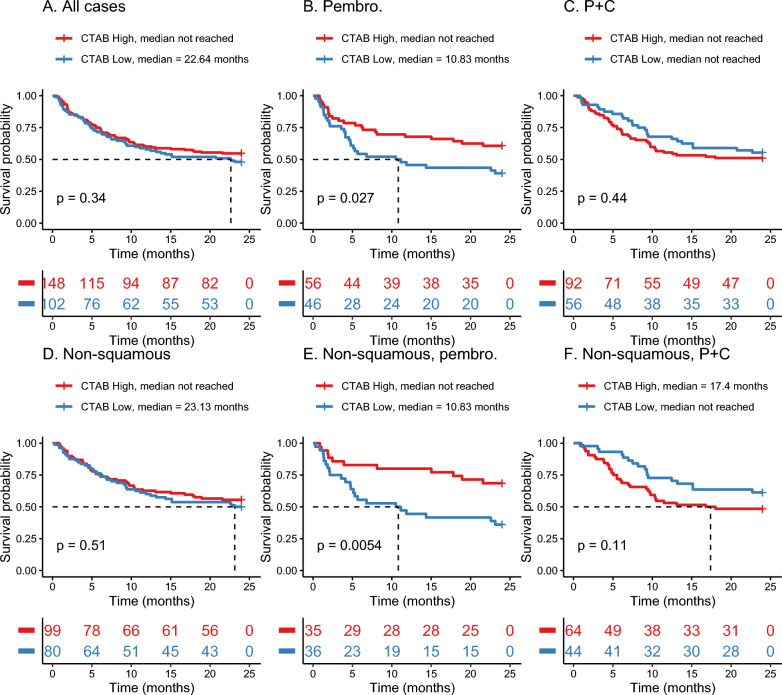
Kaplan–Meier overall survival analyses for cancer testis antigen burden (CTAB) high and low groups across various histological and treatment subgroups in the retrospective cohort: **A** All patients, **B** patients treated with pembrolizumab monotherapy, **C** patients treated with pembrolizumab in combination with chemotherapy, **D** patients with non-squamous NSCLC, **E** patients with non-squamous NSCLC treated with pembrolizumab monotherapy, **F** patients with non-squamous NSCLC treated with pembrolizumab in combination with chemotherapy. Statistical comparisons p-values indicated on each plot

To determine the relationship between CTAB and survival, we performed KM overall survival analyses on a retrospective cohort of 250 patients with NSCLC treated with anti-PD-1 immunotherapy (pembrolizumab) alone or in combination with chemotherapy (Table 2). These analyses revealed no significant difference in survival between high and low CTAB groups in the overall cohort (Fig. 4a). However, among patients treated with pembrolizumab monotherapy, the CTAB high group demonstrated significantly better survival (CTAB high: median not reached, CTAB low: median = 10.93 months; p = 0.027) (Fig. 4b). This trend was not observed among patients treated with combined pembrolizumab and chemotherapy (Fig. 4c). To evaluate histologically-derived survival differences, we divided the retrospective cohort based on histology (non-squamous vs squamous) and determined that the significant association between CTAB status and survival among the pembrolizumab monotherapy group appeared to be largely driven by non-squamous NSCLC (Fig. 4), which constituted 85% of the retrospective cohort (Table 2). In this subgroup, the significance of the relationship whereby the CTAB high group had better survival outcomes to the CTAB low group increased (p = 0.0054) (Fig. 4e).

**Fig. 5:**
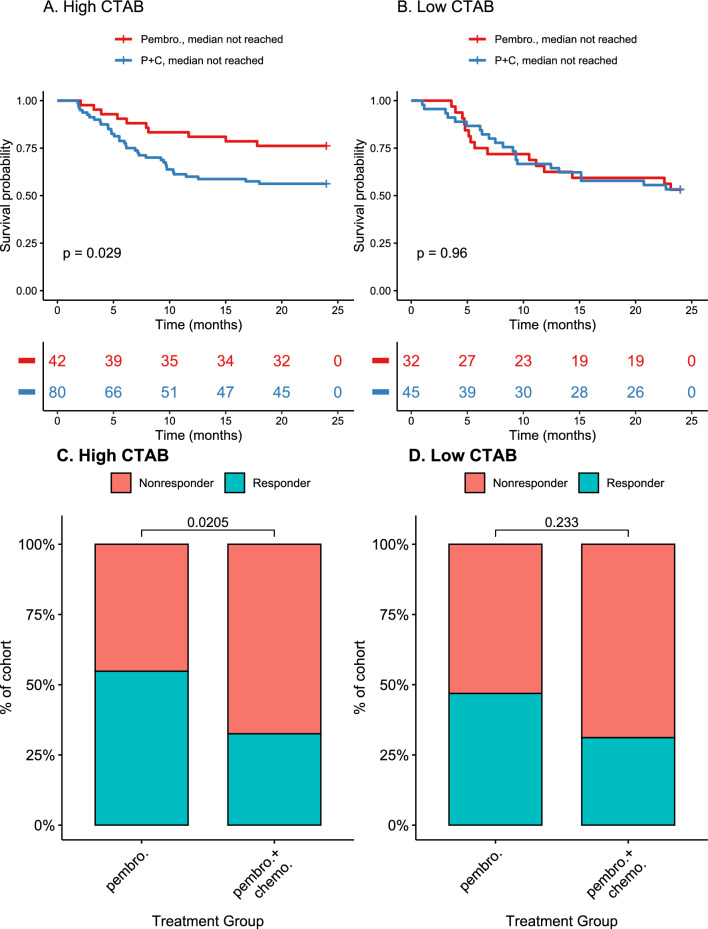
Kaplan–Meier (KM) overall survival and response rate analyses comparing treatment groups within cancer testis antigen burden (CTAB) high and low groups in the retrospective cohort: **A** KM overall survival analysis for patients with high CTAB with comparison p-value indicated, **B** KM overall survival analysis for patients with low CTAB with comparison p-value indicated, **C** response rate analysis for patients with high CTAB with Fisher’s Exact Test p-value indicated, **D** response rate analysis for patients with low CTAB with Fisher’s Exact Test p-value indicated

We performed additional KM survival analyses comparing the pembrolizumab monotherapy and combination therapy groups within the CTAB high and CTAB low groups. Within the CTAB high group, no significant difference in survival outcomes was observed between the two treatment groups (Fig. 5a). However, within the CTAB low group, the combination therapy group exhibited significantly better survival than the pembrolizumab monotherapy group (pembrolizumab monotherapy median = 10.83 months, combination therapy: median not reached; p = 0.04) (Fig. 5b). Subsequent treatment response rate analysis revealed that among patients with high CTAB, the pembrolizumab monotherapy group had a significantly greater proportion of patients responding to treatment than the combination therapy group (p = 0.02) (Fig. 5c). With the responder group only including partial or complete responses (PR or CR), this resulted in a larger proportion of patients with stable disease (SD) in the combination group as opposed to the monotherapy group (p = 0.053) (Additional file 5: Fig. S5a). Although the pembrolizumab monotherapy group also showed a higher proportion of responders among low CTAB patients and a similar distribution of stable disease patients in both treatment groups (Additional file 5: Fig. S5b), the difference was not statistically significant (Fig. 5d).

## Discussion

CTAB is a biomarker of CTA expression and co-expression that quantifies the degree to which these restrictively expressed, immunogenic antigens are present in the tumor microenvironment. The clinical utility of CTAs is well established, with evidence supporting their role as treatment targets, especially their innate immunogenicity contributing to increased response to immunotherapy.

Our initial analyses showed significant co-expression between the 17 CTAs sequenced for the tumor samples in the discovery cohort. Based on this co-expression, we developed CTAB, a biomarker capable of quantifying this co-expression by aggregating the individual gene expression ranks of these CTA genes. Applying this biomarker to the entire discovery cohort revealed a peaked distribution with a mean of 170, which was utilized as a threshold to classify the cohort into CTAB high and CTAB low groups. We subdivided the discovery cohort into 22 cancer types with variable CTA expression. Given each unique tumor type, the relative mean value of CTAB in each of the cancer types varied considerably. This suggests that some cancer types, such as melanoma, inherently had higher CTA expression, while other cancer types, such as kidney or colorectal cancer, generally exhibited lower overall CTA expression. This apparent connection between CTA expression and cancer type was particularly relevant in a treatment selection context, where treatments relying on the immunogenicity or targetability of CTA may be more applicable to some cancer types than others. This characteristic CTA expression also suggested that potential modifications or normalizations of CTAB may be possible to maximize its utility in each cancer type.

Previous work has demonstrated that single biomarker strategies often cannot capture the complexity of tumor-immune interactions, and as a result, may not be sufficient to predict response to interventions such as checkpoint inhibition, cell therapy, and cancer vaccines [36]. Given the multitude of novel immunotherapies currently being investigated and the heterogeneity of cancer, it is likely that there is no single biomarker that is predictive of every type of cancer immunotherapy [38]. One potential strategy to overcome this limitation is to assess and combine multiple biomarkers in order to develop a more complete understanding of the tumor microenvironment [36].

While investigating possible associations between CTAB and previously established solid tumor biomarkers of response to ICI therapy, CTAB was found to have no statistical relationship with PD-L1 classification, as measured by IHC. This suggests that CTAB interrogates aspects of the tumor microenvironment distinct from those assessed by PD-L1 expression alone. Similar analyses, however, revealed a significant association between TMB and CTAB whereby high TMB (TPS ≥ 10 mut/Mb) was significantly associated with higher CTAB. Though they nominally assess distinct aspects of the tumor microenvironment, the association between these two biomarkers suggests that CTAB, like TMB, is at least partially driven by cell proliferation activity. However, the development of tumor antigens in CTAB differs from TMB. CTA are proteins associated with proliferative activity expressed in cancer cells in tissues that do not normally express them, whereas the neoantigens assessed by TMB are mutated forms of many other genes expressed because of extensive cancer cell proliferation. Additionally, no significant correlation was found between CTAB and tumor inflammation as assessed by TIGS. Taken together, these results suggest that CTAB assesses microenvironmental features not entirely captured by traditional immune biomarkers like PD-L1 and TMB while still originating from phenomena associated with tumor growth and development.

Survival analyses conducted on a retrospective cohort of 250 ICI-treated patients with NSCLC revealed that among patients treated with the PD-1 inhibitor pembrolizumab alone, those with high CTAB demonstrated significantly better overall survival than those treated with pembrolizumab in combination with chemotherapy. This suggests that some patients included in the pembrolizumab monotherapy group may not be best suited to an immunotherapy-only course of treatment and indicates that the CTAB status of a patient could be an important treatment selection biomarker, since these patients have significantly worse survival when treated with immunotherapy alone. These results additionally suggest that among the low CTAB population, the chemotherapy may be the primary source of treatment efficacy, as targets of immunotherapy, as quantified by CTAB, are less prevalent among these tumors. Taken together with the lack of statistical correlation between CTAB and PD-L1 assessed by IHC, a widely used biomarker for immunotherapy response, these results suggest that CTAB may describe important additional aspects of the tumor-immune microenvironment not accounted for by traditional immunotherapy treatment selection biomarkers. Subsequent survival analyses corroborated these findings, revealing that a significant survival difference only existed between the pembrolizumab monotherapy or combination therapy groups for patients with low CTAB. Notably, these survival results were found to be even more significant when only non-squamous NSCLC was considered, suggesting that a biological difference between squamous and non-squamous NSCLC subtypes may underlie a greater role played by CTA expression in the immunotherapy-induced immune response.

To include outcome metrics other than survival in our evaluation of CTAB as a biomarker, we also considered treatment response within the retrospective cohort. Interestingly, among patients with low CTAB, a significant difference in the proportion of patients responding to treatment did not exist between the pembrolizumab monotherapy and combination therapy groups. Such a difference did however exist among patients with high CTAB whereby pembrolizumab monotherapy patients had a significantly higher proportion of responders than combination therapy patients. As the responding group in this case was taken to include only those patients exhibiting partial or complete response (PR or CR), this was found to be result of the larger proportion of patients treated with pembrolizumab and chemotherapy in combination exhibiting stable disease (SD) than those treated with pembrolizumab alone (p = 0.053, Additional file 5: Fig. S5). These results suggest that while, among patients with high CTAB, the addition of chemotherapy to pembrolizumab does not result in a significant survival difference, this change in treatment does affect the nature of the response of these patients to this drug, perhaps through the suppression of immune cell proliferation within tumors. Taken alongside the survival analyses, these results suggest that in concert with other biomarkers commonly in use for treatment selection such as PD-L1, CTAB may allow for a more nuanced identification of patient subpopulations as candidates or non-candidates for immunotherapy.

Although NGS technology is becoming standard of care for comprehensive genomic profiling to recognize tumor alterations that can predict response to immunotherapy by identifying microsatellite instability (MSI) and TMB, there are few NGS assays have been rigorously validated for use with RNA to measure cancer testes antigens as a prognostic or predictive test. Further studies within and across laboratories are needed to address access and analytical validity of CTAB measurement and to participate in larger-scale, tumor-specific clinical studies to further define and harmonize CTAB thresholds.

## Conclusions

In this work, we developed a novel pan-cancer biomarker of tumor immune microenvironment measuring CTA co-expression in solid tumors. This biomarker may potentially aid in deepening our understanding of response to cancer treatments such as immunotherapies ICIs, cell therapies and cancer vaccines. A comprehensive genomic and immune profiling strategy may benefit treatment selection as well clinical trial strategies that mitigate multitude of immune escape mechanisms driving the tumor growth. In this study, we present that in combination with other orthogonal biomarkers assessing distinct aspects of the tumor-immune microenvironment, CTAB may form an important part of a complete understanding of the tumor microenvironment and its implications for treatment. Future studies in larger, pan-cancer cohorts will be required to establish the mechanisms underlying the unique effects of CTA expression and co-expression on tumor development and treatment.

### Supplementary Information

**Additional file 1: Figure S1.** Cancer testis antigen (CTA) expression patterns across normal tissue. Normal tissue gene expression data shown was sourced from genotype-tissue expression database (GTEx).

**Additional file 2: Figure S2.** Cancer testis antigen (CTA) co-expression in the cohort compiled from The Cancer Genome Atlas (TCGA). A) Correlation plot detailing the pairwise Pearson correlations of 17 CTAs. All statistically significant (p ≤ 0.05) correlations are denoted, and an “X” indicates a nonsignificant correlation. The black rectangles about the main diagonal are visual aids indicating the three observed groups of CTA expression, and the colors of the gene labels on both axes are additional visual aids to reflect these groups. B) Network graph of CTA co-expression in the discovery cohort where the thickness and length of each edge represent the absolute value of the correlation between two CTA (a thick, short edge denotes a strong correlation and a thin, long edge denotes a weak correlation) and the color of each edge represents the direction of each observed correlation (a red edge represents a positive correlation and a blue edge represents a negative correlation). Note that edges are only shown for significant (p ≤ 0.05) correlations. The color of each node matches the axis gene labels on the correlation plot (Additional file 2: Fig. S2a) and correspond to the observed groups of CTA expression.

**Additional file 3: Figure S3.** Cancer testis antigen burden (CTAB) distributions in the cohort compiled from The Cancer Genome Atlas (TCGA): A) overall CTAB distribution (median of 196 shown), B) distributions of CTAB for 22 tumor types in the TCGA cohort.

**Additional file 4: Figure S4.** Correlation of cancer testis antigen burden (CTAB) with other biomarkers, including emerging biomarkers, in discovery cohort: A) correlation plot including Spearman correlation values, B) network diagram showing significant correlations. Correlation of CTAB with other biomarkers in the cohort compiled from The Cancer Genome Atlas (TCGA): C) correlation plot including Spearman correlation values, D) network diagram showing significant correlations.

**Additional file 5: Figure S5.** Comparison of the proportion of patients exhibiting stable disease (SD) in each of the treatment groups (pembrolizumab monotherapy and pembrolizumab combined with chemotherapy) for patients in the retrospective cohort with (A) high cancer testis antigen burden (CTAB) and (B) low CTAB. Other potential responses to treatment were one of the following Response Evaluation Criteria in Solid Tumors (RECIST) v1.1 response grades: progressive disease (PD), partial response (PR), or complete response (CR). Fisher’s Exact Test p-values indicated comparing the proportions of stable disease stable disease (SD), progressive disease (PD), partial response (PR), complete response (PR).

**Additional file 6: Table S1.** The Cancer Genome Atlas (TCGA) cohort description.

## Data Availability

The datasets generated and/or analyzed during the current study are not publicly available due to a non-provisional patent filing covering the methods used to analyze such datasets but are available from the corresponding author on reasonable request.

## Abbreviations

ICI: Immune checkpoint inhibitor
PD-1: Programmed cell death 1
CTA: Cancer testis antigen
IHC: Immunohistochemistry
PCR: Polymerase chain reaction
CTAB: Cancer testis antigen burden
PD-L1: Programmed death-ligand 1
TMB: Tumor mutational burden
NSCLC: Non-small cell lung cancer
CGIP: Comprehensive genomic and immune profiling
CLIA: Clinical Laboratory Improvement Amendments
RECIST: Response evaluation criteria in solid tumors
CR: Complete response
PR: Partial response
SD: Stable disease
PD: Progressive disease
TCGA: The Cancer Genome Atlas
GTEx: Genotype-tissue expression
FFPE: Formalin-fixed paraffin-embedded
KM: Kaplan–Meier
CP: Cell proliferation
TIGS: Tumor immunogenic score

## References

[1] Schoenfeld AJ, Hellmann MD (2020). Acquired resistance to immune checkpoint inhibitors. Cancer Cell.

[2] Darvin P, Toor SM, Sasidharan Nair V, Elkord E (2018). Immune checkpoint inhibitors: recent progress and potential biomarkers. Exp Mol Med.

[3] Vesely MD, Zhang T, Chen L (2022). Resistance mechanisms to anti-PD cancer immunotherapy. Annu Rev Immunol.

[4] Chen DS, Mellman I (2017). Elements of cancer immunity and the cancer-immune set point. Nature.

[5] Pilla L, Maccalli C (2018). Immune profiling of cancer patients treated with immunotherapy: advances and challenges. Biomedicines.

[6] Sangro B, Sarobe P, Hervás-Stubbs S, Melero I (2021). Advances in immunotherapy for hepatocellular carcinoma. Nat Rev Gastroenterol Hepatol.

[7] Dajsakdipon T, Siripoon T, Ngamphaiboon N, Ativitavas T, Dejthevaporn T (2022). Immunotherapy and biomarkers in sarcoma. Curr Treat Options Oncol.

[8] Fasano M, Corte CMD, Liello RD, Viscardi G, Sparano F, Iacovino ML (2022). Immunotherapy for head and neck cancer: present and future. Crit Rev Oncol Hematol.

[9] Mino-Kenudson M, Schalper K, Cooper W, Dacic S, Hirsch FR, Jain D (2022). Predictive biomarkers for immunotherapy in lung cancer: perspective from the international association for the study of lung cancer pathology committee. J Thorac Oncol.

[10] Monkman J, Kim H, Mayer A, Mehdi A, Matigian N, Cumberbatch M (2023). Multi-omic and spatial dissection of immunotherapy response groups in non-small cell lung cancer. Immunology.

[11] Wang Y, Zhang H, Liu C, Wang Z, Wu W, Zhang N (2022). Immune checkpoint modulators in cancer immunotherapy: recent advances and emerging concepts. J Hematol Oncol.

[12] Meng X, Sun X, Liu Z, He Y (2021). A novel era of cancer/testis antigen in cancer immunotherapy. Int Immunopharmacol.

[13] Xie K, Fu C, Wang S, Xu H, Liu S, Shao Y (2019). Cancer-testis antigens in ovarian cancer: implication for biomarkers and therapeutic targets. J Ovarian Res.

[14] Fratta E, Coral S, Covre A, Parisi G, Colizzi F, Danielli R (2011). The biology of cancer testis antigens: putative function, regulation and therapeutic potential. Mol Oncol.

[15] Nin DS, Deng LW (2023). Biology of cancer-testis antigens and their therapeutic implications in cancer. Cells.

[16] Hong S, Van Kaer L (1999). Immune privilege: keeping an eye on natural killer T cells. J Exp Med.

[17] Kalejs M, Erenpreisa J (2005). Cancer/testis antigens and gametogenesis: a review and “brain-storming” session. Cancer Cell Int.

[18] Yang P, Qiao Y, Meng M, Zhou Q (2022). Cancer/testis antigens as biomarker and target for the diagnosis, prognosis, and therapy of lung cancer. Front Oncol.

[19] Mirandola L, Cannon JM, Cobos E, Bernardini G, Jenkins MR, Kast WM (2011). Cancer testis antigens: novel biomarkers and targetable proteins for ovarian cancer. Int Rev Immunol..

[20] Grizzi F, Franceschini B, Hamrick C, Frezza EE, Cobos E, Chiriva-Internati M (2007). Usefulness of cancer-testis antigens as biomarkers for the diagnosis and treatment of hepatocellular carcinoma. J Transl Med.

[21] Jagadish N, Parashar D, Gupta N, Agarwal S, Sharma A, Fatima R (2016). A novel cancer testis antigen target A-kinase anchor protein (AKAP4) for the early diagnosis and immunotherapy of colon cancer. Oncoimmunology.

[22] Mahmoud AM (2018). Cancer testis antigens as immunogenic and oncogenic targets in breast cancer. Immunotherapy.

[23] Fan C, Qu H, Wang X, Sobhani N, Wang L, Liu S (2021). Cancer/testis antigens: from serology to mRNA cancer vaccine. Semin Cancer Biol.

[24] Thomas R, Al-Khadairi G, Roelands J, Hendrickx W, Dermime S, Bedognetti D (2018). NY-ESO-1 based immunotherapy of cancer: current perspectives. Front Immunol.

[25] Weon JL, Potts PR (2015). The MAGE protein family and cancer. Curr Opin Cell Biol.

[26] Yao J, Caballero OL, Yung WKA, Weinstein JN, Riggins GJ, Strausberg RL (2014). Tumor subtype-specific cancer-testis antigens as potential biomarkers and immunotherapeutic targets for cancers. Cancer Immunol Res.

[27] Zhang Y, Zhang Y, Zhang L (2019). Expression of cancer-testis antigens in esophageal cancer and their progress in immunotherapy. J Cancer Res Clin Oncol.

[28] Conroy JM, Pabla S, Glenn ST, Burgher B, Nesline M, Papanicolau-Sengos A (2018). Analytical validation of a next-generation sequencing assay to monitor immune responses in solid tumors. J Mol Diagn JMD.

[29] Conroy JM, Pabla S, Glenn ST, Seager RJ, Van Roey E, Gao S (2021). A scalable high-throughput targeted next-generation sequencing assay for comprehensive genomic profiling of solid tumors. PLoS ONE.

[30] Nesline MK, Previs RA, Dy GK, Deng L, Lee YH, DePietro P (2023). PD-L1 expression by RNA-sequencing in non-small cell lung cancer: concordance with immunohistochemistry and associations with pembrolizumab treatment outcomes. Cancers.

[31] Gao GF, Parker JS, Reynolds SM, Silva TC, Wang LB, Zhou W (2019). Before and after: comparison of legacy and harmonized TCGA genomic data commons’ data. Cell Syst.

[32] GTEx Consortium (2013). The genotype-tissue expression (GTEx) project. Nat Genet..

[33] Fruchterman TMJ, Reingold EM (1991). Graph drawing by force-directed placement. Softw Pract Exp.

[34] Pabla S, Conroy JM, Nesline MK, Glenn ST, Papanicolau-Sengos A, Burgher B (2019). Proliferative potential and resistance to immune checkpoint blockade in lung cancer patients. J Immunother Cancer.

[35] Zhang T, Pabla S, Lenzo FL, Conroy JM, Nesline MK, Glenn ST (2020). Proliferative potential and response to nivolumab in clear cell renal cell carcinoma patients. Oncoimmunology.

[36] Pabla S, Seager RJ, Van Roey E, Gao S, Hoefer C, Nesline MK (2021). Integration of tumor inflammation, cell proliferation, and traditional biomarkers improves prediction of immunotherapy resistance and response. Biomark Res.

[37] Forrester JV, Xu H, Lambe T, Cornall R (2008). Immune privilege or privileged immunity?. Mucosal Immunol.

[38] McKean WB, Moser JC, Rimm D, Hu-Lieskovan S (2020). Biomarkers in precision cancer immunotherapy: promise and challenges. Am Soc Clin Oncol Educ Book Am Soc Clin Oncol Annu Meet.

