# Supplementary figures and images for: Cancer testis antigen burden (CTAB): a novel biomarker of tumor-associated antigens in lung cancer
Source: J Transl Med. 2024 Feb 7;22:141. doi: 10.1186/s12967-024-04918-0 (PMC10851610; doi:10.1186/s12967-024-04918-0)

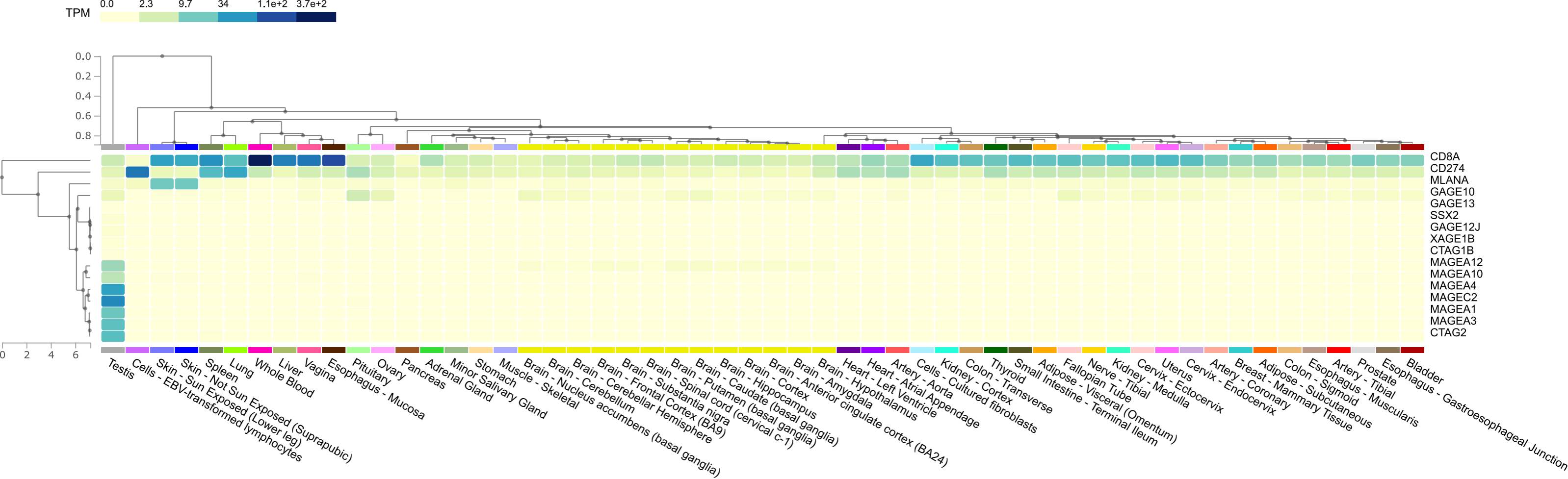

Supplement: Supplementary file 1 — Additional file 1: Figure S1. Cancer testis antigen (CTA) expression patterns across normal tissue. Normal tissue gene expression data shown was sourced from genotype-tissue expression database (GTEx). [file 12967_2024_4918_MOESM1_ESM.pdf]

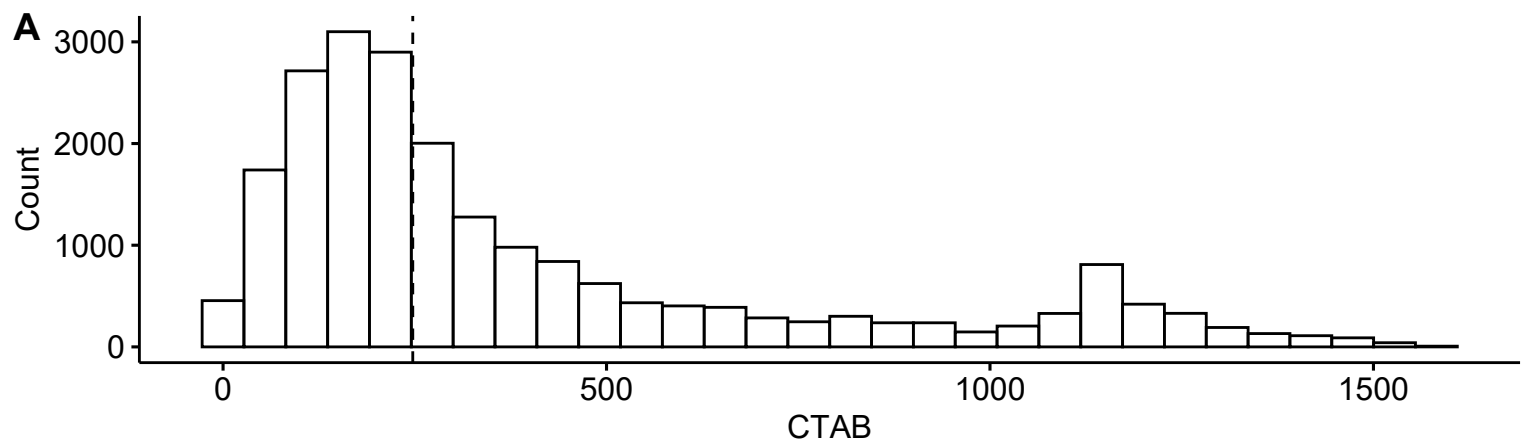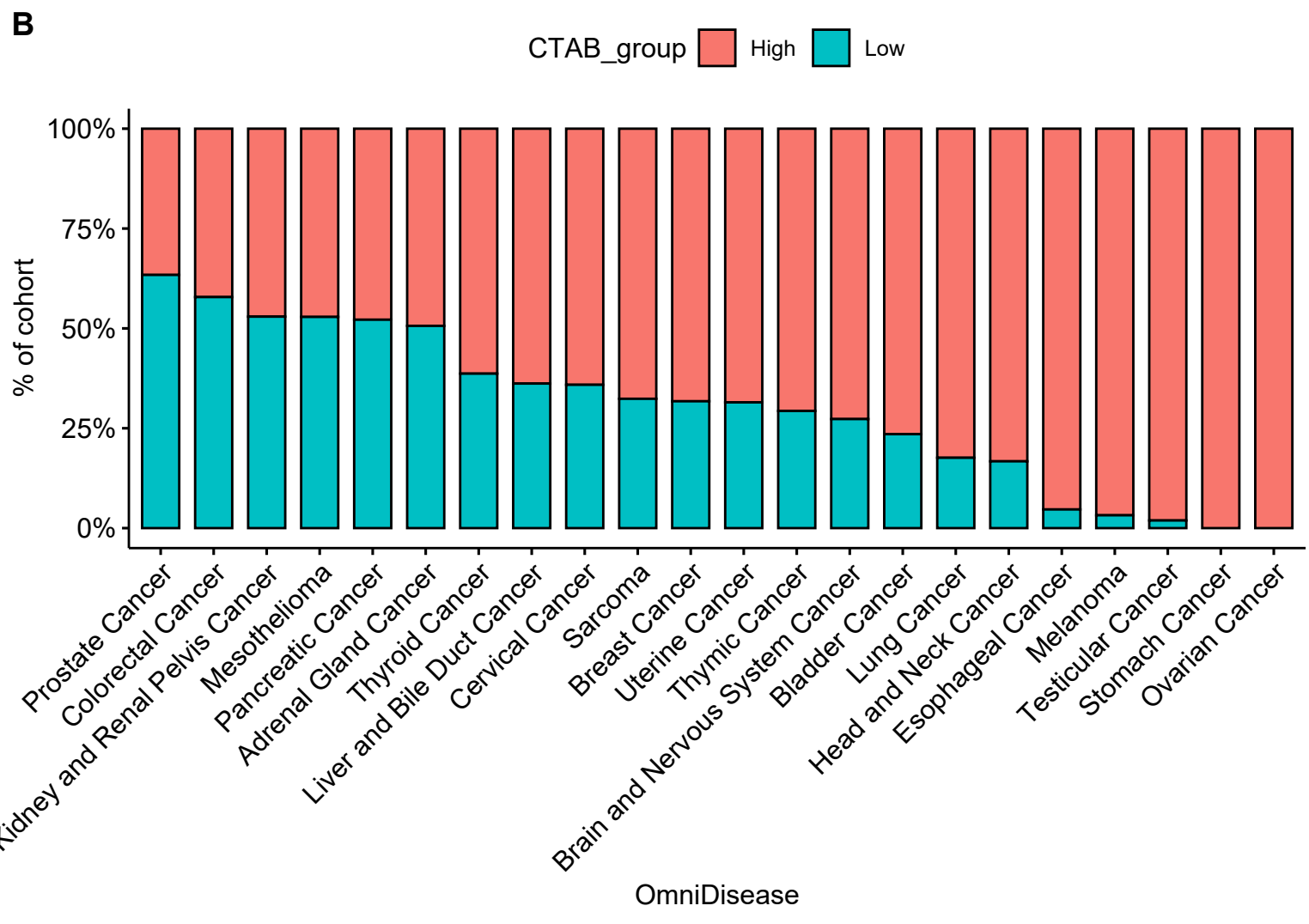

Supplement: Supplementary file 3 — Additional file 3: Figure S3. Cancer testis antigen burden (CTAB) distributions in the cohort compiled from The Cancer Genome Atlas (TCGA): A) overall CTAB distribution (median of 196 shown), B) distributions of CTAB for 22 tumor types in the TCGA cohort. [file 12967_2024_4918_MOESM3_ESM.pdf]

## A. High CTAB

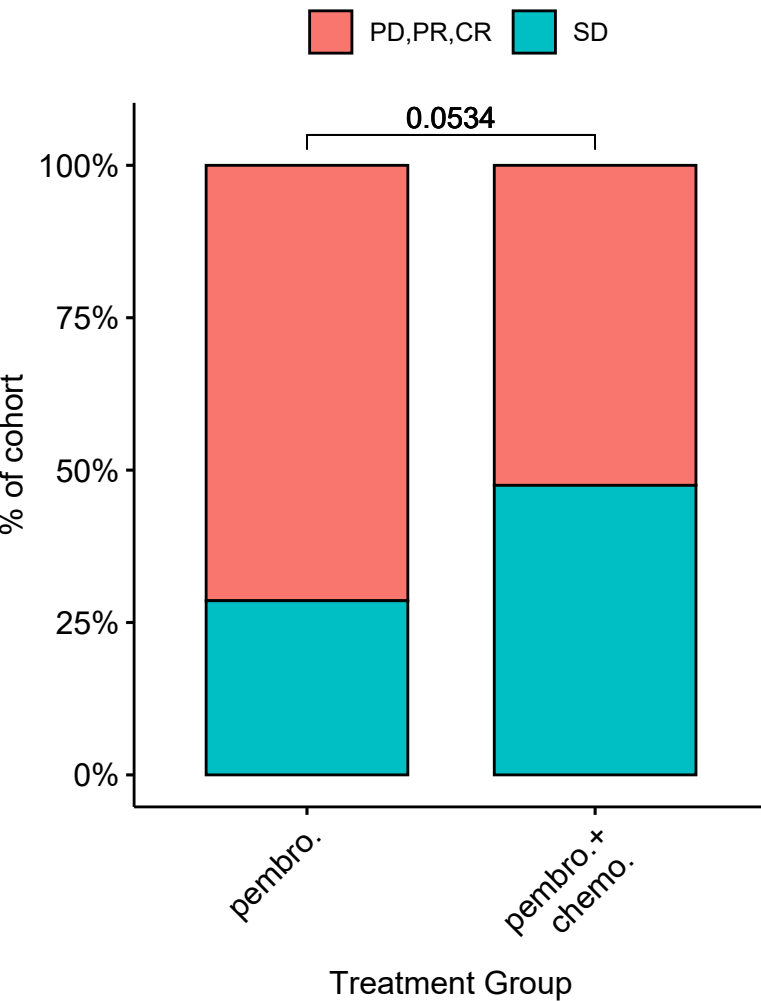

## B. Low CTAB

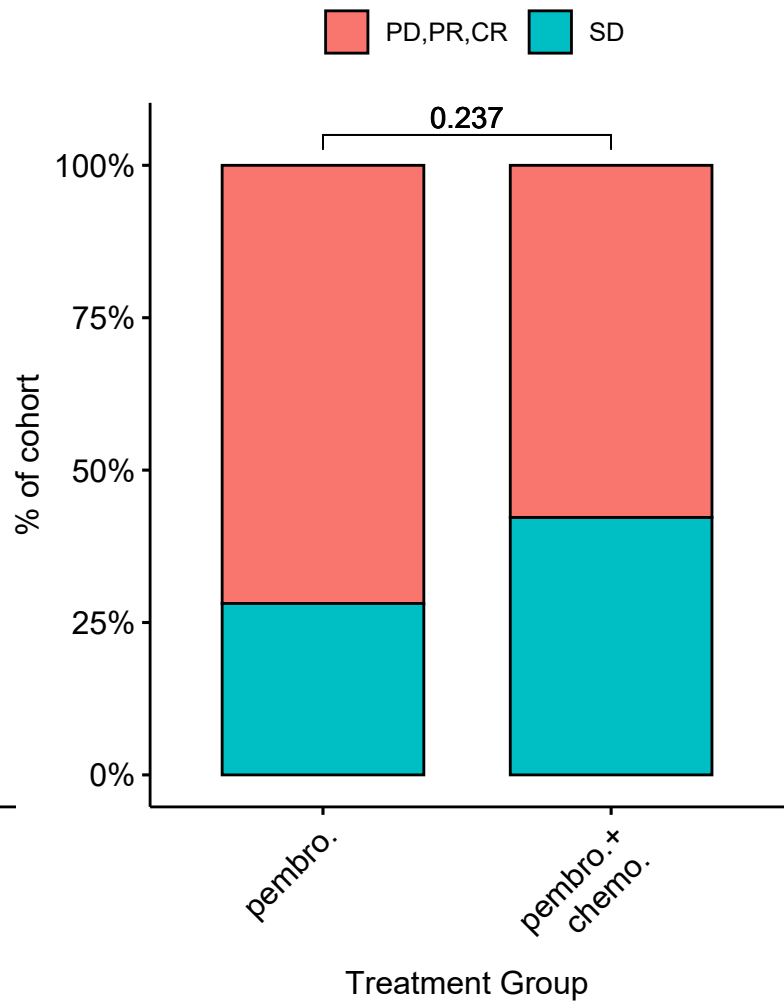

Supplement: Supplementary file 5 — Additional file 5: Figure S5. Comparison of the proportion of patients exhibiting stable disease (SD) in each of the treatment groups (pembrolizumab monotherapy and pembrolizumab combined with chemotherapy) for patients in the retrospective cohort with (A) high cancer testis antigen burden (CTAB) and (B) low CTAB. Other potential responses to treatment were one of the following Response Evaluation Criteria in Solid Tumors (RECIST) v1.1 response grades: progressive disease (PD), partial response (PR), or complete response (CR). Fisher’s Exact Test p-values indicated comparing the proportions of stable disease stable disease (SD), progressive disease (PD), partial response (PR), complete response (PR). [file 12967_2024_4918_MOESM5_ESM.pdf]
