# Supplementary material for: Cancer testis antigen burden (CTAB): a novel biomarker of tumor-associated antigens in lung cancer
Source: J Transl Med. 2024 Feb 7;22:141. doi: 10.1186/s12967-024-04918-0 (PMC10851610; doi:10.1186/s12967-024-04918-0)

A

	MLANA	GAGE10	GAGE13	GAGE2	GAGE1	GAGE12J	MAGEA10	MAGEA4	CTAG1B	CTAG2	MAGEA12	MAGEC2	MAGEA1	MAGEA3	SSX2	BAGE	XAGE1B
MLANA	1.00	-0.07	0.04	0.03	0.05	0.03	X	-0.04	-0.03	0.04	-0.03	0.02	0.04	0.02	X	0.09	-0.02
GAGE10	-0.07	1.00	0.22	0.12	0.16	0.14	0.05	X	0.06	0.04	0.09	0.02	X	X	0.10	0.07	X
GAGE13	0.04	0.22	1.00	0.56	0.58	0.54	0.28	0.23	0.25	0.28	0.25	0.29	0.30	0.29	0.19	0.27	0.23
GAGE2	0.03	0.12	0.56	1.00	0.64	0.66	0.35	0.33	0.35	0.41	0.35	0.43	0.43	0.43	0.25	0.34	0.35
GAGE1	0.05	0.16	0.58	0.64	1.00	0.71	0.36	0.30	0.34	0.38	0.33	0.41	0.40	0.39	0.26	0.35	0.32
GAGE12J	0.03	0.14	0.54	0.66	0.71	1.00	0.37	0.35	0.38	0.41	0.37	0.43	0.45	0.44	0.26	0.36	0.35
MAGEA10	X	0.05	0.28	0.35	0.36	0.37	1.00	0.46	0.31	0.33	0.31	0.33	0.38	0.41	0.17	0.25	0.25
MAGEA4	-0.04	X	0.23	0.33	0.30	0.35	0.46	1.00	0.34	0.35	0.33	0.33	0.46	0.52	0.07	0.18	0.28
CTAG1B	-0.03	0.06	0.25	0.35	0.34	0.38	0.31	0.34	1.00	0.63	0.36	0.38	0.39	0.42	0.16	0.25	0.27
CTAG2	0.04	0.04	0.28	0.41	0.38	0.41	0.33	0.35	0.63	1.00	0.38	0.43	0.44	0.46	0.24	0.29	0.32
MAGEA12	-0.03	0.09	0.25	0.35	0.33	0.37	0.31	0.33	0.36	0.38	1.00	0.45	0.41	0.54	0.18	0.30	0.26
MAGEC2	0.02	0.02	0.29	0.43	0.41	0.43	0.33	0.33	0.38	0.43	0.45	1.00	0.53	0.56	0.27	0.35	0.39
MAGEA1	0.04	X	0.30	0.43	0.40	0.45	0.38	0.46	0.39	0.44	0.41	0.53	1.00	0.59	0.21	0.33	0.36
MAGEA3	0.02	X	0.29	0.43	0.39	0.44	0.41	0.52	0.42	0.46	0.54	0.56	0.59	1.00	0.21	0.31	0.40
SSX2	X	0.10	0.19	0.25	0.26	0.26	0.17	0.07	0.16	0.24	0.18	0.27	0.21	0.21	1.00	0.24	0.21
BAGE	0.09	0.07	0.27	0.34	0.35	0.36	0.25	0.18	0.25	0.29	0.30	0.35	0.33	0.31	0.24	1.00	0.27
XAGE1B	-0.02	X	0.23	0.35	0.32	0.35	0.25	0.28	0.27	0.32	0.26	0.39	0.36	0.40	0.21	0.27	1.00

B

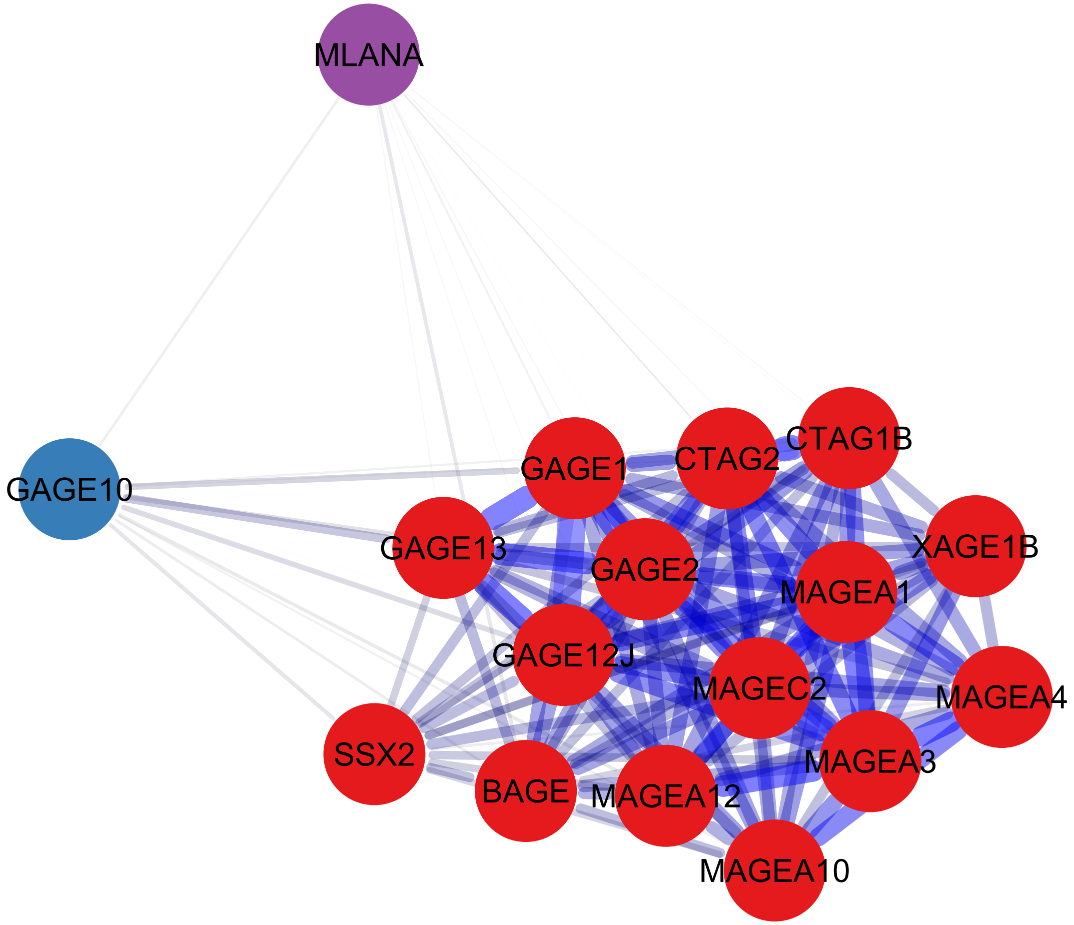

Supplement: Supplementary file 2 — Additional file 2: Figure S2. Cancer testis antigen (CTA) co-expression in the cohort compiled from The Cancer Genome Atlas (TCGA). A) Correlation plot detailing the pairwise Pearson correlations of 17 CTAs. All statistically significant (p ≤ 0.05) correlations are denoted, and an “X” indicates a nonsignificant correlation. The black rectangles about the main diagonal are visual aids indicating the three observed groups of CTA expression, and the colors of the gene labels on both axes are additional visual aids to reflect these groups. B) Network graph of CTA co-expression in the discovery cohort where the thickness and length of each edge represent the absolute value of the correlation between two CTA (a thick, short edge denotes a strong correlation and a thin, long edge denotes a weak correlation) and the color of each edge represents the direction of each observed correlation (a red edge represents a positive correlation and a blue edge represents a negative correlation). Note that edges are only shown for significant (p ≤ 0.05) correlations. The color of each node matches the axis gene labels on the correlation plot (Additional file 2: Fig. S2a) and correspond to the observed groups of CTA expression. [file 12967_2024_4918_MOESM2_ESM.pdf]
