# Supplementary material for: Cancer testis antigen burden (CTAB): a novel biomarker of tumor-associated antigens in lung cancer
Source: J Transl Med. 2024 Feb 7;22:141. doi: 10.1186/s12967-024-04918-0 (PMC10851610; doi:10.1186/s12967-024-04918-0)

A

	PD-L1 (IHC)	TIGS	PD-L1 (RNA)	CTAB	CP	TMB
PD-L1 (IHC)	1.00	0.29	0.61	~~0.09~~	0.03	0.10
TIGS	0.29	1.00	0.63	~~0.02~~	-0.22	~~-0.07~~
PD-L1 (RNA)	0.61	0.63	1.00	~~0.06~~	-0.03	0.04
CTAB	~~0.09~~	~~0.02~~	~~0.06~~	1.00	0.03	0.07
CP	0.03	-0.22	-0.03	0.03	1.00	0.08
TMB	0.10	~~-0.07~~	0.04	0.07	0.08	1.00

B

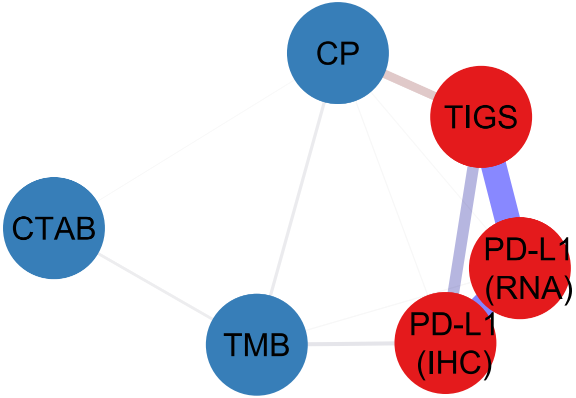

C

	TIGS	PD-L1 (RNA)	CTAB	CP
TIGS	1.00	0.57	0.09	0.12
PD-L1 (RNA)	0.57	1.00	0.07	0.03
CTAB	0.09	0.07	1.00	0.40
CP	0.12	0.03	0.40	1.00

D

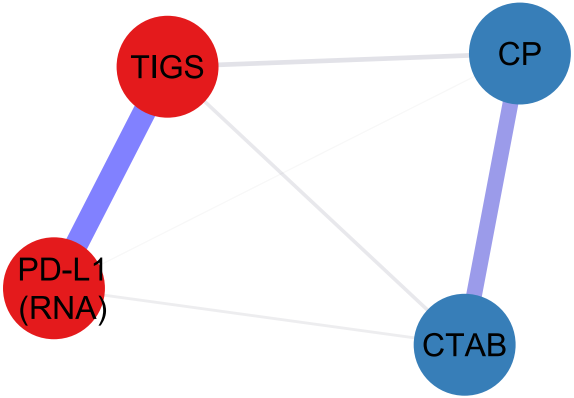

Supplement: Supplementary file 4 — Additional file 4: Figure S4. Correlation of cancer testis antigen burden (CTAB) with other biomarkers, including emerging biomarkers, in discovery cohort: A) correlation plot including Spearman correlation values, B) network diagram showing significant correlations. Correlation of CTAB with other biomarkers in the cohort compiled from The Cancer Genome Atlas (TCGA): C) correlation plot including Spearman correlation values, D) network diagram showing significant correlations. [file 12967_2024_4918_MOESM4_ESM.pdf]
