# Supplementary material for: Cancer testis antigen burden (CTAB): a novel biomarker of tumor-associated antigens in lung cancer
Source: J Transl Med. 2024 Feb 7;22:141. doi: 10.1186/s12967-024-04918-0 (PMC10851610; doi:10.1186/s12967-024-04918-0)
Supplement: Supplementary file 6 — Additional file 6: Table S1. The Cancer Genome Atlas (TCGA) cohort description. [file 12967_2024_4918_MOESM6_ESM.docx]

**Table S1.** The Cancer Genome Atlas (TCGA) cohort description.

| **Variable** | **Group** | **N** | **% of Cohort** |
| --- | --- | --- | --- |
| **Age** | [10,20) | 95 | 0.4% |
|  | [20,30) | 743 | 3.4% |
|  | [30,40) | 1573 | 7.2% |
|  | [40,50) | 2942 | 13.4% |
|  | [50,60) | 4758 | 21.7% |
|  | [60,70) | 6066 | 27.6% |
|  | [70,80) | 4231 | 19.3% |
|  | [80,90) | 1352 | 6.2% |
|  | [90,100) | 164 | 0.8% |
|  | No Data | 51 | 0.2% |
| **Sex** | Female | 11333 | 51.6% |
|  | Male | 10642 | 48.4% |
| **Cancer Type** | Adrenal Gland Cancer | 79 | 0.4% |
|  | Bladder Cancer | 693 | 3.2% |
|  | Brain and Nervous System Cancer | 1315 | 6.0% |
|  | Breast Cancer | 3045 | 13.9% |
|  | Cervical Cancer | 379 | 1.7% |
|  | Colorectal Cancer | 1390 | 6.3% |
|  | Esophageal Cancer | 364 | 1.7% |
|  | Head and Neck Cancer | 1196 | 5.4% |
|  | Kidney and Renal Pelvis Cancer | 2854 | 13.0% |
|  | Liver and Bile Duct Cancer | 1387 | 6.3% |
|  | Lung Cancer | 2697 | 12.3% |
|  | Melanoma | 529 | 2.4% |
|  | Mesothelioma | 87 | 0.4% |
|  | Ovarian Cancer | 365 | 1.7% |
|  | Pancreatic Cancer | 253 | 1.2% |
|  | Prostate Cancer | 1292 | 5.9% |
|  | Sarcoma | 349 | 1.6% |
|  | Stomach Cancer | 898 | 4.1% |
|  | Testicular Cancer | 209 | 1.0% |
|  | Thymic Cancer | 150 | 0.7% |
|  | Thyroid Cancer | 1510 | 6.9% |
|  | Uterine Cancer | 934 | 4.3% |
| **All Samples** | | 21975 | 100.0% |
